# Factors influencing optimal postoperative pain management: a systematic scoping review

**DOI:** 10.3389/fpain.2026.1870836

**Published:** 2026-07-29

**Authors:** Raeesah Fakier, Romy Parker, Mitchell Sarkies, Katleho Limakatso

**Affiliations:** 1 Pain Management Unit, Department of Anaesthesia and Perioperative Medicine, Neuroscience Institute, University of Cape Town, Cape Town, South Africa; 2 Sydney School of Health Services, Faculty of Medicine and Health, University of Sydney, Sydney, NSW, Australia; 3 Agency for Clinical Innovation NSW, St Leonards, NSW, Australia

**Keywords:** barriers, enablers, pain management, postoperative pain, theoretical domains framework

## Abstract

Effective postoperative pain management is a critical component of surgical care, directly influencing patient recovery, satisfaction, and long-term outcomes. Despite recent advances in pain management, postoperative pain remains poorly managed worldwide, suggesting that postoperative pain outcomes are influenced by several factors beyond the inherent efficacy of available treatments. The aim of this scoping review was to map the factors influencing postoperative pain management and to generate interventions that support optimal, evidence-based pain management after surgery. Articles published until September 2025 were identified through a systematic search of the following electronic databases: PubMed (MEDLINE) via EBSCOhost, CINAHL (Cumulative Index to Nursing & Allied Health Literature) via EBSCOhost, and PsycINFO via EBSCOhost. Two reviewers independently conducted the screening of articles and data extraction. Barriers and enablers were synthesized using the Theoretical Domains Framework. Intervention strategies were generated by mapping the identified barriers onto the Capability, Opportunity, Motivation-Behavior model. Patient empowerment and active engagement in pain management, strong clinician-patient rapport, and structured, team-based and policy-driven support were identified as key enablers for optimizing postoperative pain management. On the other hand, a lack of knowledge and misconception about postoperative pain, capability gaps in assessing and managing pain, interprofessional and communication challenges, organizational and policy deficiencies, and resource and workforce constraints were key barriers to optimal postoperative pain management. Despite advancements in treatment options, the findings suggest that optimal pain management requires a multifaceted approach that addresses both individual and systemic challenges. These insights contribute to a deeper understanding of the barriers and enablers of effective pain management and highlight the need for targeted interventions across various levels of healthcare delivery.

## 1 Introduction

Effective postoperative pain management is a fundamental component of high-quality surgical care and plays a critical role in shaping both short- and long-term patient outcomes (1). Adequate control of postoperative pain facilitates early mobilization, reduces the risk of complications, and contributes to faster functional recovery following surgery (2). Conversely, poorly managed postoperative pain is associated with a range of adverse consequences, including increased morbidity, prolonged hospitalization, and greater healthcare utilization (3). Importantly, inadequate acute postoperative pain control is associated with chronic postoperative pain, a condition that significantly impairs the quality of life and function long after the initial surgical intervention (4).

Despite substantial advances in perioperative pain management strategies over the past two decades, postoperative pain remains a critical challenge worldwide. A recent meta-analysis revealed that 58% (95% CI 36.8–76.7) of individuals who have undergone surgery experience moderate-severe pain within two weeks after hospital discharge (5). The Enhanced Recovery After Surgery (ERAS) protocols were introduced to facilitate improvements in surgical outcomes including postoperative pain (6). However, desirable postoperative pain outcomes are yet to be achieved consistently across health systems (7). The persistence of uncontrolled postoperative pain suggests that pain management is influenced by a range of contextual factors that are beyond the inherent efficacy of available treatments.

The current evidence highlights patient, clinician, and health system-related factors that influence postoperative pain management. For example, patient knowledge about the surgical procedure, postoperative pain experience, and options for pain management (8), clinician capability in assessing pain and administering treatment (9), and institutional policies and protocols for pain management, have been shown to have an effect on pain outcomes after surgery (10). These determinants may function as barriers that impede effective pain control or as enablers that support evidence-based pain management practices.

A growing body of research has sought to identify these barriers and enablers within different clinical contexts. However, there has been no comprehensive synthesis of the barriers and enablers influencing postoperative pain management within hospital settings. Synthesizing the existing evidence can help guide the design of contextually appropriate interventions, inform policy, support the implementation of clinical guidelines aimed at strengthening perioperative pain management. Therefore, the aim of this review was to systematically synthesize the existing literature on the barriers and enablers influencing postoperative pain management within hospital settings and generate interventions for improvement.

## 2 Methods

### 2.1 Study design

This scoping review was conducted in accordance with the Joanna Briggs Institute (JBI) manual for evidence synthesis (11) and reported according to the Preferred Reporting Items for Systematic Reviews and Meta-Analyses extension for Scoping review guidelines (PRISMA-ScR) (12). The protocol for this review was registered on Open Science Framework and has since been published (13).

We utilized the Theoretical Domains Framework (TDF) (14) and the Capability, Opportunity, Motivation-Behavior (COM-B) model (15) as guiding frameworks. The TDF is a comprehensive implementation science framework that synthesizes 33 behaviour change theories to identify factors influencing implementation and decision-making in healthcare and other settings (16). It consists of 14 domains, each containing several constructs. The identified barriers were mapped onto the TDF. The COM-B model serves as a higher-level model that synthesizes and organizes TDF domains into broader components needed for behavior change (17). The TDF and COM-B model were identified as suitable frameworks for this scoping review because they have been validated to systematically assess barriers and enablers to behaviour change in healthcare settings (18). In addition, they are widely used to design interventions for improving clinical practice and outcomes (19), aligning with the review's objective to generate interventions for improving postoperative pain management.

### 2.2 Search strategy

A customized search strategy (Supplementary File S1), including relevant terms and phrases related to enablers (e.g., facilitator), barriers (e.g., challenges), and postoperative pain (e.g., postsurgical pain), was used to identify eligible studies published from a journal's inception up to September 2025 from the following databases: PubMed (MEDLINE) via EBSCOhost, CINAHL (Cumulative Index to Nursing & Allied Health Literature) via EBSCOhost, and PsycINFO via EBSCOhost.

We considered primary studies that utilized qualitative, quantitative, and mixed methods designs to identify enablers and barriers to optimal postoperative pain management of adults in a hospital setting. Enablers were defined as factors that enhance postoperative pain management, while barriers were defined as those that hinder it. These factors are categorized into three groups: patient, clinician, and system-related factors. An intervention was defined as an action designed to affect behaviour that would lead to improved pain control and relief after surgery. We excluded studies on postoperative pain management in paediatric patients due to variations between treatment guidelines for adults and children (20). In addition, we excluded grey literature, including preprints and conference proceedings, due to a lack of editorial scrutiny associated with these publications (21). Owing to a lack of translation resources, only articles published in English were included.

### 2.3 Study selection and data extraction

The identified studies were exported to Covidence—a web-based tool for managing literature reviews (https://www.covidence.org), which facilitated study de-duplication, screening, and data extraction. Two reviewers (R.F & K.L) independently screened study titles and abstracts using the eligibility criteria. Potentially eligible studies were assessed for eligibility in full-text form by the same reviewers. A customised, pre-piloted data extraction tool was used for data extraction. Variables that were recorded include author names, year of study publication, country of origin and economic status, study design, participant characteristics (e.g., participant type: patients or health care worker), sample size, barriers, and enablers.

### 2.4 Data synthesis

NVivo data management software was utilized to facilitate data analysis on the enablers and barriers to optimal postoperative pain management (22). Enablers and barriers were synthesized using inductive thematic analysis, whereby conceptually similar items were iteratively grouped based on shared meaning and underlying constructs (23). Themes were refined through repeated comparison and consensus among the research team to ensure coherence, clarity, and analytic rigor. Barriers were mapped across the relevant domains of the TDF (Supplementary File S2) (16). The TDF constructs were mapped onto the COM-B model to generate possible intervention strategies (15). Quantitative data analysis included content analysis to map the frequency of identified enablers and barriers and a comparative analysis of outcome trends between high income countries (HICs) and low- and middle-income countries (LMICs).

## 3 Results

**Figure 1:**
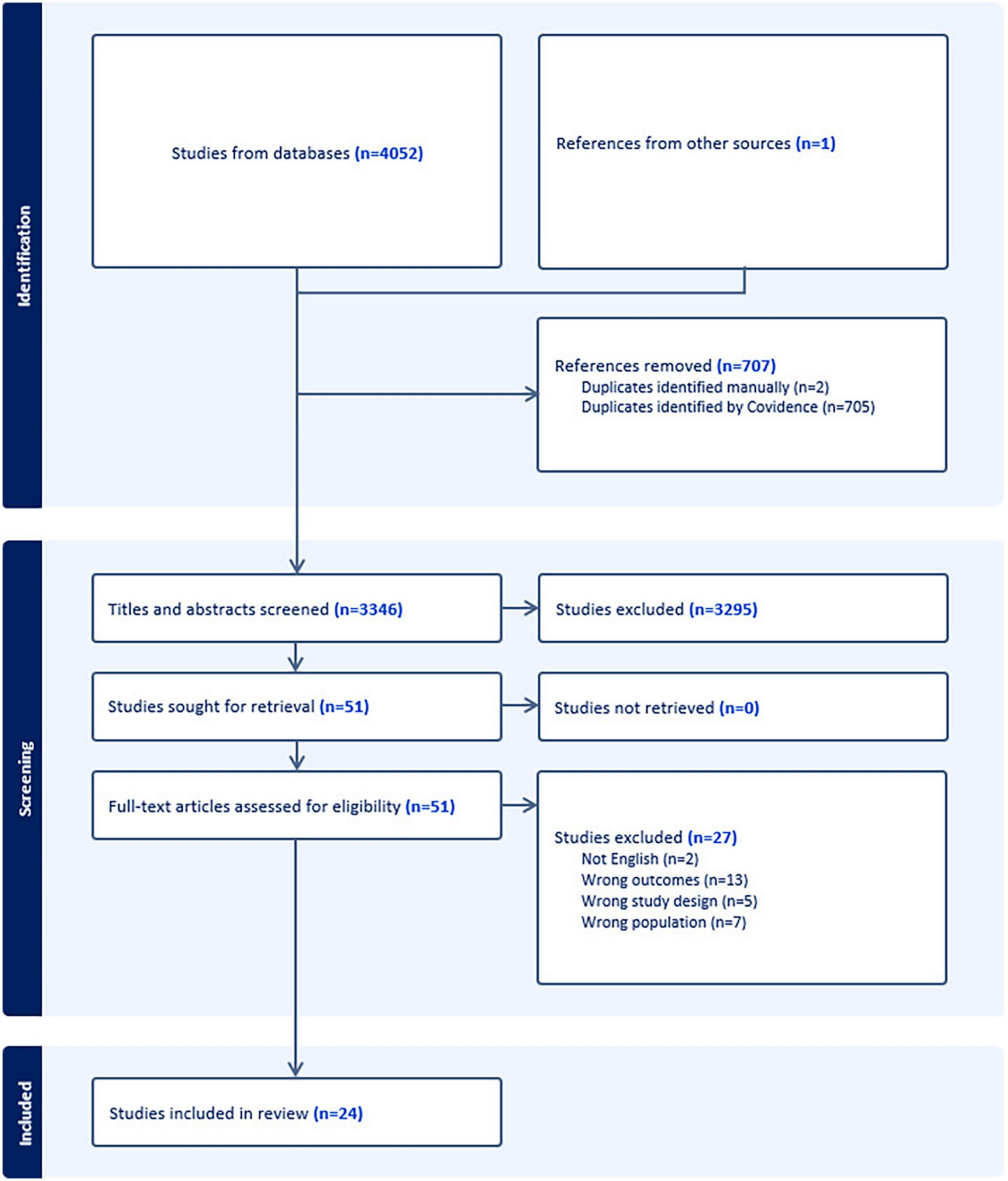
The PRISMA flow chat.

The screening process is illustrated in Figure 1. The literature search identified 4,053 studies, and 3,346 were retained after de-duplication. Following the screening of titles and abstracts, 51 studies were deemed eligible for full-text review. Of these, 24 studies met the eligibility criteria and were included in this review. The screening of titles and abstracts and full-text articles reflected moderate (0.68) and strong (0.82) inter-rater agreement, respectively.

### 3.1 Study characteristics

**Figure 2:**
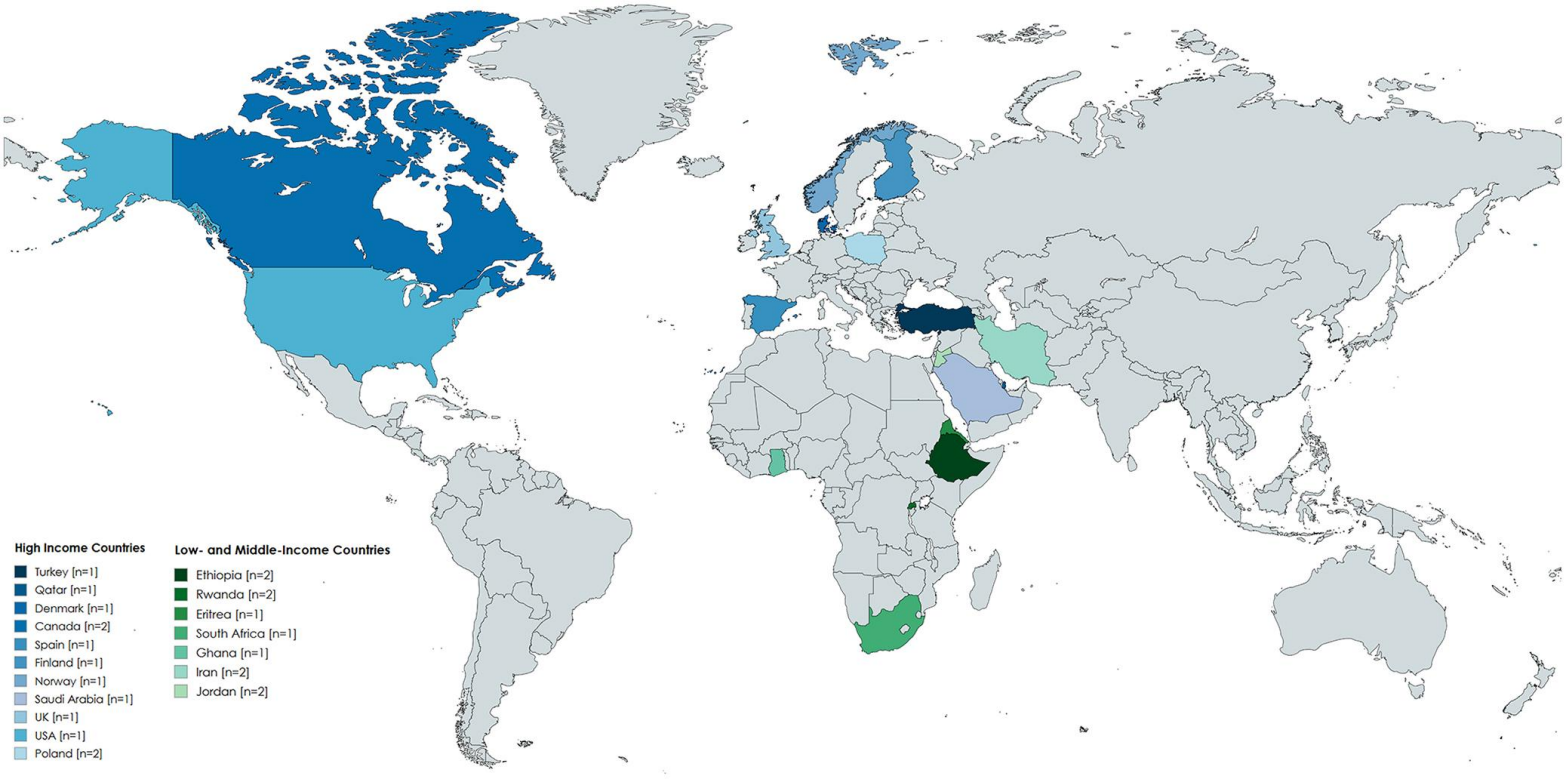
Income status of countries in which included studies were conducted.

The study characteristics are summarized in Supplementary Table S1. Ten studies used a descriptive qualitative design (24–33), four used a qualitative cross-sectional design (8, 34–36), five used a mixed-methods design (37–41), and five used a quantitative cross-sectional survey (9, 10, 42–44). Eleven studies were conducted in seven LMICs and 13 studies were conducted in 11 HICs (Figure 2). The included studies were published between 2001 and 2026.

### 3.2 Participants characteristics

Included studies provided data from a total of 5,383 participants. These included 814 patients and 4,569 health care professionals involved in postoperative care, the majority of whom were nurses (*n* = 4303). Other health care professionals (*n* = 266) included surgeons, physiotherapists, general practitioners, anesthetists, gynecologists, dentists, and healthcare assistants. Given the dominant representation of nurses in this review, findings relating to clinicians should be interpreted primarily as reflecting the perspectives of nurses.

### 3.3 Enablers and barriers to effective postoperative pain management

Thematic data analysis generated 14 themes from the identified enablers and barriers. The frequency of each theme across the included studies is presented in Table 1. Differences in theme frequency between HICs and LMICs were observed only for patient-related barriers: fear and negative perceptions of pain medication, and individual and contextual limitations affecting pain assessment and management. For both themes, a greater number of studies were conducted in HICs than in LMICs. No notable differences were identified across the remaining themes.

**Table 1:** The frequency of enabler- and barrier-related themes across HICs and LMICs.

Category	Theme	Number of studies reporting theme [*n* (%)]	Studies in HICs [*n* = x/13]	Studies in LMICs [*n* = x/11]
Enablers				
Patient-related	Empowerment and active involvement in pain management^a^	3 (13)	1 (24)	2 (26, 31)
Clinician-related (predominantly nurses)	Positive clinician–patient rapport^a^	2 (8)	-	2 (26, 31)
System-related	Structured, team-based, and policy-driven pain management support^a^	3 (13)	1 (24)	2 (26, 31)
Barriers				
Patient-related	Communication and pain expression challenges	9 (38)	4 (28, 37, 39, 42)	5 (10, 26, 29, 33, 44)
	Fear and negative perceptions of pain medication	9 (38)	6 (28, 34, 37, 39, 40, 42)	3 (26, 33, 44)
	Limited knowledge and misconceptions about pain and analgesia	6 (25)	3 (8, 24, 34)	3 (26, 32, 33)
	Individual and contextual limitations affecting pain assessment and management	5 (21)	4 (8, 9, 37, 39)	1 (33)
Clinician-related (predominantly nurses)	Knowledge and competency gaps in pain assessment and management	10 (42)	5 (9, 24, 25, 39, 42)	5 (10, 27, 38, 43, 44)
	Cross-discipline communication and collaboration challenges	6 (25)	3 (25, 37, 42)	3 (27, 32, 43)
	Misconceptions about post-operative pain	5 (21)	3 (24, 37, 40)	2 (10, 43)
	Professional constraints	5 (21)	3 (8, 9, 37)	2 (26, 31)
System-related	Organizational and policy deficiencies	15 (63)	9 (8, 9, 24, 30, 35–37, 39, 42)	6 (10, 31, 32, 41, 43, 44)
	Resource and workforce constraints	10 (42)	5 (35, 36, 39, 40, 42)	5 (10, 27, 38, 41, 43)
	Care coordination and continuity challenges^a^	4 (17)	4 (8, 25, 30, 37)	-

a These themes are underpinned by limited evidence (<5 studies) and should be interpreted with caution.

#### 3.3.1 Patient-related enablers

##### 3.3.1.1 Empowerment and active engagement in pain management

Effective postoperative pain management was facilitated when patients were empowered through the provision of comprehensive pre- and postoperative information regarding the surgical procedure, expected pain experiences, and available pain management options (26). Freedom to choose analgesic modalities and administration intervals (24), combined with willingness to express and report pain (31), supported shared decision-making and timely pain control. Clinicians’ respect for patient rights and adherence to professional ethical standards further strengthened postoperative pain management (31).

#### 3.3.2 Clinician-related enablers (predominantly nurses)

##### 3.3.2.1 Positive clinician–patient rapport

Positive clinician–patient rapport emerged as a key enabler, fostering trust, open communication, and accurate pain reporting (26). Clinicians’ cultural competence in engaging patients from diverse linguistic and cultural backgrounds enhanced understanding of pain experiences and promoted patient-centered pain management (26).

#### 3.3.3 System-related enablers

##### 3.3.3.1 Structured, team-based, and policy-driven pain management support

System-level facilitation of postoperative pain management was evident in the availability of in-service training and online educational resources, supervision of trainees, and implementation of standardized pain management guidelines and policies (26, 31). A coordinated, multidisciplinary team approach further supported consistent, high-quality pain management across care settings (31).

#### 3.3.4 Patient-related barriers

##### 3.3.4.1 Limited knowledge and misconceptions about pain and analgesia

A limited understanding of the surgical procedure, expected postoperative pain, and available pain management options were prominent barriers (8, 24, 26, 32, 33). Some patients questioned the effectiveness of prescribed analgesics, particularly oral medications compared with intravenous administration (24, 34), and believed that analgesics were unnecessary during tissue healing (26).

##### 3.3.4.2 Fear and negative perceptions of pain medication

Patients expressed fear of addiction (26, 28, 33, 34, 37, 39, 44), dependence (28, 33, 39), drug abuse (44), and adverse side effects of analgesics (34). Additional apprehensions included fear of injections (40) and fear to request pain medication (42).

##### 3.3.4.3 Communication and pain expression challenges

Barriers to pain communication included reluctance to report pain (33, 39, 42, 44) and a tendency to report pain only to physicians rather than nursing staff (29, 37, 42). Language barriers (10, 28, 29, 37), cultural and religious beliefs discouraging pain expression (26, 29, 39), social praise for enduring pain (26, 39), and limited pain expression among older adults (28) further impeded accurate pain assessment and timely intervention.

##### 3.3.4.4 Individual and contextual limitations affecting pain assessment and management

Pain management was additionally hindered by cognitive impairment (37), hearing or visual impairment (39), and difficulty completing self-report pain assessment tools (37). Environmental factors, such as sleep disruption due to noisy wards (8), preoperative fasting restrictions (9), and a lack of familiarity with clinical staff (33), further compromised effective pain control.

#### 3.3.5 Clinician-related barriers (predominantly nurses)

##### 3.3.5.1 Misconceptions about postoperative pain

Clinicians perceived postoperative pain as an unavoidable or poorly modifiable consequence of surgery, leading to its low prioritization in care (24, 43). Misconceptions included disbelief in patient-reported pain (37), reliance on observable pain behaviors to guide assessment (40), and the assumption that non-pharmacological interventions are ineffective after surgery (10, 24). Some clinicians also viewed analgesia administration as a task-oriented responsibility rather than part of a holistic pain management approach (10).

##### 3.3.5.2 Knowledge and competency gaps in pain assessment and management

Limited specialized knowledge and inadequate proficiency in administering pain assessment tools and treatment contributed to inconsistent pain evaluation and management (9, 10, 29, 38, 39, 42–44). Clinicians demonstrated limited awareness of existing pain management protocols (27) and frequently failed to conduct comprehensive pain assessments or routinely evaluate treatment effectiveness (25).

##### 3.3.5.3 Cross-discipline communication and collaboration challenges

Pain management was further compromised by poor clinician–patient communication (27, 42) and weak interprofessional collaboration (43). Inconsistent sharing of pain-related information (25), physicians’ distrust of nursing pain assessments (37), difficulty accepting patient pain reports (37, 43), and inadequate cooperation among nurses, physicians, and pharmacists hindered coordinated and timely pain management (43). Poor clinician–patient rapport further limited effective pain discussions (32).

##### 3.3.5.4 Professional constraints

Work-related fatigue (8) and discomfort prescribing analgesia in the context of inadequate monitoring reduced proactive pain management (9). Fear of legal repercussions related to opioid misuse and adverse effects (26, 37), powerlessness to intervene beyond prescribed orders (31), and failure to document pain management plans contributed to sub-optimal and fragmented care.

#### 3.3.6 System-level barriers

##### 3.3.6.1 Organizational and policy deficiencies

Postoperative pain management was hindered by the absence of standardized approaches across surgical procedures (24) and a lack of local policies or clinical guidelines (9, 10, 30, 31, 35–37, 39, 41–43). Pain management was often not prioritized at an institutional level, reflected in under-reporting of postoperative pain outcomes (8), limited administrative support (10), unclear instructions regarding analgesic administration (39), and restricted nursing authority within physician-centered models of care (31, 32). Limited access to specialist pain services further constrained evidence-based management (44).

##### 3.3.6.2 Resource and workforce constraints

Inadequate resources significantly affected pain management delivery. High patient-to-clinician ratios, staff shortages, heavy workloads, and insufficient time to implement non-pharmacological interventions reduced clinicians’ capacity to manage pain effectively. Limited availability of analgesics, particularly narcotics and opioids due to high cost (10, 27, 35, 36, 38–43), along with equipment shortages (40), and a lack of physical and psychological self-management strategies (42) further compromised pain control.

##### 3.3.6.3 Care coordination and continuity challenges

Fragmentation of care was evident in disruptions to pain management continuity from the recovery room to ward settings (8, 30). Limited opportunities for multidisciplinary discussion of pain management plans (37) and inconsistent communication among care team members (25) impeded coordinated decision-making and timely adjustments to analgesic strategies.

### 3.4 Interventions for behavior change

The intervention strategies for behavior change and intervention examples are summarized in Table 2. A total of 68 barriers were mapped to 11 TDF domains and linked to all three COM-B components. The Behavior Change Wheel was then used to map COM-B components to intervention functions, as defined in Supplementary File S3. For example, *limited competency in conducting comprehensive pain assessment* was coded to the TDF domain “skills”, reflecting a deficit in clinicians’ competence in performing pain assessments. Within the COM-B model, this was mapped to psychological capability, as the barrier relates to insufficient knowledge and skill to execute the behavior effectively. Based on established Behavior Change Wheel linkages, this mapping informed the selection of the intervention function “training”, as structured skills development is required to improve competence in comprehensive pain assessment.

**Table 2:** Mapping of barriers onto the TDF and COM-B.

Barrier statement	Frequency of mention	TDF domain	Construct	COM-B	Intervention function	Intervention example
Patient-related barriers						
Limited knowledge about the surgical procedure, postoperative pain experience, and options for pain management	6	Knowledge	Knowledge	Psychological Capability	Education	Structured pre-operative pain education sessions with written and visual materials
Perceived ineffectiveness of medication administered per orally vs. intravenously.	1	Beliefs about Consequences	Outcome expectancies	Reflective motivation	Persuasion	Clinician-led explanation comparing oral and IV analgesic efficacy
Requesting or taking medication in anticipation of future pain.	1	Beliefs about Consequences	Outcome expectancies	Reflective motivation	Education	Teaching appropriate timing and use of analgesics
Nil Per OS restrictions	1	Environmental Context and Resources	Physical constraints	Physical Opportunity	Environmental restructuring	Prescribing alternative non-oral analgesic routes
Fear of addiction to pain medication	4	Beliefs about Consequences	Outcome expectancies	Reflective motivation	Persuasion	Reassurance using evidence-based opioid risk education
Fear of adverse side-effects	2	Beliefs about Consequences	Outcome expectancies	Reflective motivation	Persuasion	Counselling on side-effect monitoring and management
Concerns about ineffectiveness of prescribed analgesics	1	Beliefs about Consequences	Outcome expectancies	Reflective motivation	Persuasion	Sharing expected pain relief timelines
Lack of adequate sleep overnight due to noisy wards.	1	Environmental Context and Resources	Environmental stressors	Physical Opportunity	Environmental restructuring	Implementing quiet-time policies
Social praise for “handling pain well”	2	Social Influences	Social pressure	Social Opportunity	Modelling	Staff modelling open pain reporting as acceptable
Belief analgesics are unnecessary during tissue healing	1	Beliefs about Consequences	Outcome expectancies	Reflective Motivation	Education	Education on benefits of pain control for recovery
Language barrier with staff	4	Environmental Context and Resources	Barriers and facilitators	Physical Opportunity	Environmental restructuring	Access to interpreters or translated pain tools
Uncooperative behaviour with pain medication	1	Behavioural regulation	Breaking habit	Psychological Capability	Enablement	Individualised support and negotiated pain plans
Limited pain expression in older adults.	1	Social/Professional Role and Identity	Social identity	Reflective Motivation	Enablement	Proactive pain assessment using age-appropriate tools
Fear of dependence and addiction to pain medication	3	Beliefs about Consequences	Outcome expectancies	Reflective Motivation	Education	Structured education on short-term vs. long-term opioid use
Cultural and religious beliefs hindering pain expression.	2	Social Influences	Social norms	Social Opportunity	Modelling	Culturally sensitive clinician communication demonstrating acceptance of pain reporting
Reporting pain only to doctors	3	Social/professional role and identity	Beliefs about professional roles and boundaries	Reflective Motivation	Education	Clarifying nurses’ role in pain assessment and management
Reluctance to report pain	4	Emotion	Fear	Automatic Motivation	Enablement	Reassurance and repeated invitation to report pain
Reluctance to take pain medication	1	Beliefs about Consequences	Outcome expectancies	Reflective Motivation	Persuasion	Addressing misconceptions regarding harm and dependency
Fear to ask for pain medication	2	Emotion	Fear/anxiety	Automatic Motivation	Enablement	Reassurance and routine pain enquiry by staff
Difficulty in completing self-report pain assessment forms and scales.	1	Skills	Ability	Physical Capability	Training	Simplified pain scales with staff assistance
Difficulty assessing pain in patients with cognitive impairments.	1	Environmental context and resources	Barriers and facilitators	Physical Opportunity	Environmental restructuring	Use of observational pain assessment tools
Hearing and/or visual impairment in the elderly	1	Environmental context and resources	Barriers and facilitators	Physical opportunity	Environmental restructuring	Use of large-print tools and verbal explanations
Fear of injections	1	Emotion	Fear	Automatic Motivation	Enablement	Offering alternative formulations (liquids, patches)
Aversion to taking tablets	1	Emotion	Negative affect	Automatic Motivation	Enablement	Offering liquid, transdermal, or alternative formulations
Not personally known by clinical staff	1	Social Influences	Social support	Social Opportunity	Environmental restructuring	Assigning consistent nursing staff where feasible
Clinician-related barriers (predominantly nurses)						
The misconception that post-surgical pain cannot be managed effectively, regardless of the quality of pain management.	1	Beliefs about Consequences	Beliefs	Reflective Motivation	Education	Audit and feedback showing improved pain outcomes
The belief that non-pharmacological treatments are ineffective after surgery.	2	Beliefs about Consequences	Beliefs	Reflective Motivation	Education	Training on evidence-based non-pharmacological strategies
Concerns about the addictive potential of opioids commonly used for postoperative pain management	6	Beliefs about Consequences	Outcome expectancies	Reflective Motivation	Education	Training on opioid stewardship and safe prescribing
Limited proficiency using pain assessment tools.	2	Skills	Competence	Psychological Capability	Training	Hands-on workshops using validated pain scales
Discomfort prescribing analgesia due to insufficient monitoring	1	Beliefs about consequences	Organisational culture	Reflective Motivation	Enablement	Improved monitoring systems and prescribing support
Lack specialised knowledge and skills in pain assessment and management.	10	Knowledge	Procedural knowledge	Psychological Capability	Training	Mandatory pain management competency programs
Lack of empathy towards patient suffering.	3	Emotion	Fear	Automatic Motivation	Modelling	Senior clinicians modelling empathic pain care
Fatigue due to work overload	1	Environmental Context and Resources	N/A^a^	Physical Opportunity	Environmental restructuring	Adjusted staffing and shift scheduling
Inconsistent sharing of pain-related information among clinicians	1	Social Influences	N/A	Social Opportunity	Environmental restructuring	Standardised handover and documentation tools
Limited competency in conducting comprehensive pain assessment	1	Skills	Competence	Psychological Capability	Training	Case-based pain assessment training
Lack of routine evaluation of treatment efficacy.	1	Behavioural Regulation	Self-monitoring	Psychological Capability	Enablement	Mandatory reassessment protocols
Fear of legal consequences due to adverse side effects and opioid dependency.	2	Emotion	Fear	Automatic Motivation	Enablement	Clear medico-legal guidance and institutional support
Poor communication with patients regarding pain management	2	Skills	Interpersonal skills	Psychological Capability	Training	Communication skills training focused on pain dialogue
Limited hands-on experience in pain management	2	Skills	Skills development	Physical Capability	Training	Supervised clinical exposure to pain management
Inconsistent approaches to postoperative pain assessment.	2	Memory, attention and decision processes	Organisational culture	Psychological Capability	Environmental restructuring	Standardised pain assessment protocols
Lack of awareness of existing pain management protocols.	1	Knowledge	Procedural knowledge	Psychological Capability	Education	Orientation sessions on pain guidelines
Belief that a nurse’s primary task is to administer pain relief medication rather than contribute to a more holistic approach to pain management.	1	Social/Professional Role and Identity	Role boundaries	Reflective Motivation	Persuasion	Role clarification through professional development
Physicians’ distrust in nursing pain assessments.	1	Social Influences	N/A	Social Opportunity	Modelling	Joint nurse–physician pain rounds
Difficulty believing patient pain reports.	2	Beliefs about Consequences	N/A	Reflective Motivation	Education	Training on subjective nature of pain
Inadequate cooperation and support from physicians and pharmacists.	1	Social Influences	N/A	Social Opportunity	Environmental restructuring	Multidisciplinary pain management meetings
Failure to prioritise pain management, viewing it as a normal part of surgery	1	Goals	N/A	Reflective Motivation	Persuasion	Leadership messaging prioritising pain as a vital sign
Failure to document the patient’s pain treatment plan	1	Skills	Skills assessment	Physical Capability	Training	Documentation training with audit feedback
Powerlessness to intervene independently, limited to following doctor’s orders.	1	Social/Professional Role and Identity	Professional boundaries	Reflective Motivation	Enablement	Nurse-initiated analgesia protocols
Poor clinician-patient rapport.	1	Social Influences	N/A	Social Opportunity	Modelling	Role modelling effective therapeutic communication
Failure to assess pain based on the misconception that a patient not displaying pain-related behaviours is not in pain.	1	Beliefs about Consequences	N/A	Reflective Motivation	Education	Education on non-verbal and subjective pain indicators
System-related factors						
Absence of a standardised approach to postoperative pain management across surgical procedures.	1	Environmental Context and Resources	Organisational culture	Physical Opportunity	Environmental restructuring	Implementing hospital-wide post-op pain pathways
A lack of local policies/guidelines on effective pain management	10	Environmental context and resources	Resources/material resources	Physical Opportunity	Environmental restructuring	Development and dissemination of pain guidelines
Lack of in-service training on effective pain management after surgery	3	Skills	Skills development	Psychological Capability	Training	Regular in-service pain education sessions
Under-reporting of postoperative pain outcomes.	1	Behavioural regulation	self-monitoring	Psychological Capability	Enablement	Mandatory pain score documentation in charts
Disruption in continuity of pain management from the recovery room to the wards.	3	Environmental Context and Resources	Organisational culture	Physical Opportunity	Environmental restructuring	Standardised pain handover from theatre/PACU to wards
A high patient-to-clinician ratio	1	Environmental Context and Resources	Resources/material resources	Physical Opportunity	Environmental restructuring	Workforce planning to reduce ratios
Limited narcotics and opioids availability due to high cost	1	Environmental Context and Resources	Resources/material resources	Physical Opportunity	Environmental restructuring	Budget allocation for essential analgesics
Postoperative pain not prioritised in healthcare.	1	Social/Professional Role and identity	Leadership	Reflective Motivation	Persuasion	Executive-led prioritisation of pain outcomes
Limited resources for pain management (medication, equipment, staff)	10	Environmental Context and Resources	Resources/material resources	Physical Opportunity	Environmental restructuring	Dedicated budget allocation for pain services
Lack of administrative support	1	Environmental Context and Resources	Organisational culture	Physical Opportunity	Environmental restructuring	Management endorsement of pain initiatives
Heavy workload	4	Environmental Context and Resources	Environmental stressors	Physical Opportunity	Environmental restructuring	Redistribution of workload and task delegation
Staff shortages	1	Environmental Context and Resources	Resources/material resources	Physical Opportunity	Environmental restructuring	Strategic recruitment and retention plans
Lack of alternative comfort measures	1	Environmental Context and Resources	Barriers and facilitators	Physical Opportunity	Environmental restructuring	Provision of heat/cold packs, positioning aids, etc
Inadequate time to conduct non-pharmacological pain treatments.	2	Environmental Context and Resources	Barriers and facilitators	Physical Opportunity	Environmental restructuring	Protected time for non-pharmacological-based interventions
Limited opportunity to discuss the patient’s pain management with the care team.	1	Environmental Context and Resources	Organisational culture	Physical Opportunity	Environmental restructuring	Scheduled multidisciplinary pain rounds
Unclear instructions regarding the administration of requested analgesics.	1	Knowledge	Procedural knowledge	Psychological Capability	Education	Clear prescribing and administration protocols
Limited nursing authority due to physician-centred practice	1	Social/Professional Role and Identity	Professional boundaries	Reflective Motivation	Enablement	Policy-supported nurse-led analgesia pathways
Limited access to professional pain specialists.	1	Environmental Context and Resources	Resources/material resources	Physical Opportunity	Environmental restructuring	Establishing referral pathways to pain services

a N/A indicates that no existing Theoretical Domains Framework construct sufficiently aligned with the identified barrier.

The intervention functions span education, persuasion, environmental restructuring, modelling, enablement, and training. The most common intervention strategies for patient-related barriers are enablement, environmental restructuring, and education. The most common intervention strategies for clinician-related barriers (predominantly nurses) are education and training. The most common intervention strategies for system-level barriers are environmental restructuring and enablement.

## 4 Discussion

This scoping review systematically synthesized the literature on the enablers and barriers to optimal postoperative pain management. Patient empowerment and active engagement in pain management, positive clinician-patient rapport, and structured, team-based, and policy-driven support were identified as key enablers for optimizing postoperative pain management. On the other hand, limited knowledge and misconceptions about pain, fear and negative perceptions of pain medication, communication and pain expression challenges, individual and contextual limitations, knowledge and competency gaps, cross-discipline communication and collaboration challenges, professional constraints, organizational and policy deficiencies, resource and workforce constraints, and care coordination and continuity challenges, were key barriers to optimal postoperative pain management.

Limited knowledge about the surgical procedure, postoperative pain experience, and options for pain management was identified as the most common patient-related barrier across the included studies, despite growing efforts to advocate for patient preoperative pain education over the past two decades (45). A recent exploratory study of individuals with limb amputations highlighted preoperative education as a key priority to help them prepare for surgery and manage life demands and expectations postoperatively (46). This priority is not only based on patient preferences but is also supported by evidence that demonstrates positive outcomes. For instance, a recent literature review demonstrated that preoperative patient education interventions were associated with low postoperative pain severity, reduced opioid use, and improved psychological outcomes, particularly anxiety (47). A recent nation-wide study evaluating a perioperative pain management bundle in South Africa identified patient education as the most effective element for improving postoperative pain (48). In addition, several trials have shown clinically superior postoperative pain outcomes in individuals who received preoperative education compared to those who did not (49–51), underscoring the importance of education as a prerequisite to perioperative care. Interestingly, one study included in this review identified that providing comprehensive information about the surgical procedure, postoperative pain experience, and options for pain management was an enabler for optimizing postoperative pain management (26). Together with COM-B-generated interventions in this review, this evidence supports the implementation of preoperative education as a crucial strategy for improving postoperative pain outcomes.

Concern about the addictive potential of opioids was a common barrier among both patients and clinicians in this review. While patients had concerns about long-term opioid dependence (28, 33, 39), clinicians were hesitant to prescribe opioids for severe pain due to fear of legal ramifications associated with opioid-related morbidity and mortality (26, 37). These findings are supported by those of a qualitative enquiry of 20 physicians and specialists, which revealed they were hesitant to prescribe opioids due to the fear of imprisonment, losing their medical license, or being labeled as an over prescriber (52). In that study, some clinicians argued that the professional risks associated with prescribing opioids significantly outweigh the potential benefits for patients, thereby impacting the quality of patient care. While the benefits of short-term opioid use postoperatively are well-established (53), it is imperative for physicians and surgeons to strictly adhere to safe opioid prescribing practice guidelines (54) and leverage effective, non-pharmacological treatments that are relatively safe for both short- and long-term use (55).

We found it surprising that capability deficits in pain assessment and management were the most reported barriers among clinicians, the majority of whom were nurses. Despite the critical role that nurses (who constitute 94% of the clinicians in this review) and other healthcare professionals have in managing postoperative pain, this finding highlights a significant gap in the necessary knowledge and skills required for effective pain management. Pain has been widely promoted as the fifth vital sign, reflecting the expectation that clinicians should routinely assess and manage it with the same rigor as other physiological parameters (56). However, the integration of pain as a fifth vital sign has not been universally accepted in clinical practice. In one study (57), 77% of nurses were unaware of pain being designated as a fifth vital sign, and 57% were opposed to evaluating it as such due to a heavy workload, lack of time, staffing shortages, and the perception that patients would voluntarily report pain when they experienced it.

Contrary to this idea, our findings indicate that individuals may be reluctant to report pain for various reasons, including not wanting to burden the nursing staff (39, 42). Furthermore, several studies found that patients were more likely to report pain to doctors than to nurses, despite nurses having more frequent contact with patients (29, 37, 42). Limited awareness of the importance of systematic pain assessment, coupled with conflicting attitudes toward its prioritization, may be an artefact of commonly seen inconsistencies in postoperative pain documentation, variability in assessment intervals and methods, and ultimately suboptimal pain management and monitoring (41). Targeted, ongoing training focused on knowledge and skills transfer is essential to ensure healthcare staff are equipped with the necessary expertise to assess and manage postoperative pain effectively, ultimately improving patient outcomes and standardizing care practices.

System-related barriers including limited resources for pain management such as opioid analgesics and a lack of local policies and guidelines on effective pain management, were common across HICs and LMICs. However, a striking imbalance exists in opioid use—the *Lancet* Commission on global access to palliative care and pain relief revealed that 90% of distributed morphine-equivalent opioids is consumed in the top 10% of HICs, whereas the poorest 50% of the world's population has access to only 1% of opioids distributed annually (58). This highlights equity issues, likely driven by market barriers, strict regulations, and broader historical and social influences that restrict access in LMICs (59). To this end, the World Health Organization (WHO) published a guideline to support LMICs in ensuring safe, equitable, and affordable access to essential controlled medicines (60).

Although internationally recognised institutions and organizations such as the National Institute for Health and Care Excellence (NICE) and International Association for the Study of Pain (IASP) develop guidelines that are widely adopted globally, it is not uncommon to have context-specific guidelines and policies that guide clinical practice at a local level. For example, in line with the recommendations for enhancing recovery after surgery (ERAS), Chen et al. (61) developed clinical practice guidelines for postoperative pain management in China based on the evidence that variations in cultural differences, healthcare resources, the availability of pain medications, and health policies warrant different approaches for managing postoperative pain. In the same way, Aziato and Adejumo (62) developed a context appropriate clinical guideline for postoperative pain management in Ghana. In failing to consider local factors that affect pain management, generalized approaches may not meet the unique needs of diverse patient populations. On the other hand, carefully addressing these factors by introducing local guidelines, policies, and innovative models of care that leverage existing resources, may ensure equitable and effective patient-centred care after surgery. Pagano et al. (63) provide guidance on reaching agreement when developing local perioperative pathways, highlighting key social and cognitive processes underpinning consensus. In a related study (64), they also outline practical criteria to guide the adoption of clinical recommendations. Together, these findings offer practical guidance for implementing guidelines and models of care at a local level.

### 4.1 Strengths and limitations

According to our knowledge, this is the first review to systematically synthesize the literature on the factors influencing postoperative pain management. We leveraged robust implementation sciences frameworks to highlight key areas of concern and to generate intervention strategies for optimized postoperative pain care. In addition, although patients were underrepresented in this review, the evidence for patient-related barriers was derived from both direct patient reports and clinician observations, enabling triangulation across data sources. However, a notable limitation of this review is the overrepresentation of nurses relative to other professional groups, suggesting that most identified barriers and enablers are specific to this clinician group and may not fully reflect the experiences of other healthcare professionals. It is important to interpret these results within this context. The exclusion of non-English studies may have resulted in the omission of relevant evidence, particularly from LMICs. The predominance of qualitative studies within the included literature may limit the generalizability of the findings across settings. Although formal methodological quality appraisal is not typically undertaken in scoping reviews, the absence of a quality assessment limits interpretation of the robustness and strength of the available evidence. Several themes identified in this review were supported by fewer than five studies, indicating a limited evidence base. As such, these findings should be interpreted with caution. We were unable to perform *a priori* time series analysis to identify temporal trends in outcomes due to the skewed distribution of publication years. Most included studies were published within the last decade, with only a limited number of studies from earlier years, thus precluding a meaningful comparison across time periods. The marked increase in publications over the past decade may reflect the growing recognition and application of implementation science frameworks within healthcare. We recommend that future research explore whether there have been improvements in reported barriers to postoperative pain management and investigate the mechanisms driving such changes.

## 5 Conclusion

This review highlights the complex interplay of patient, clinician, and system-related factors in postoperative pain management. Despite advances in treatment options, optimal postoperative pain management requires a multifaceted approach that addresses both individual and systemic challenges. While interventions such as education are effective at addressing individual-level barriers by enhancing patient understanding and clinician capability, further research should focus on developing and implementing innovative models of care that leverage existing resources to address system-related barriers, particularly in LMICs.
