# Supplementary material for: Factors influencing optimal postoperative pain management: a systematic scoping review
Source: Front Pain Res (Lausanne). 2026 Jul 29;7:1870836. doi: 10.3389/fpain.2026.1870836 (PMC13463247; doi:10.3389/fpain.2026.1870836)
Supplement: Supplementary file 1 [file Table1.docx]

Supplementary Table S1: A summary of study and participant characteristics and outcomes

| Authors | Country | Economic status | Design | Aim | Sample [size] | Barriers | Enablers |
| --- | --- | --- | --- | --- | --- | --- | --- |
| Bilik et al., 2018 | Turkey | HIC^[1]^ | Qualitative descriptive | To investigate orthopaedic nurses’ experiences in pain management. | Orthopaedic nurses [n=16] | Patient-related:   - Limited knowledge about the surgical procedure, postoperative pain experience, and options for pain management. - Perceived ineffectiveness of medication administered per orally versus intravenously. - Requesting or taking medication in anticipation of future pain.   Clinician-related:   - The misconception that postoperative pain cannot be managed effectively, regardless of the quality of pain management. - The belief that nonpharmacological treatments are ineffective after surgery. - Concerns about the addictive potential of opioids commonly used for postoperative pain management. - Limited proficiency in using various pain assessment tools.   System-related:   - The absence of a standardised approach to postoperative pain management across surgical procedures. | Patient-related:   - Freedom to choose pain medication and administration intervals   System-related:   - In-service training and access to online material on pain management. |
| Bouri et al., 2018 | Qatar | HIC | Quantitative cross-sectional survey | To investigate orthopaedic professionals’ recognition and knowledge of pain and perceived barriers to optimal pain management at five hospitals | Surgeons, nurses, & physiotherapists [n=361] | Patient-related:   - Not allowed to consume any food or fluids orally (Nil Per OS)   Clinician-related:   - Physicians' discomfort with prescribing pain medication due to insufficient monitoring. - Limited specialised knowledge and skills in pain assessment and management.   System-related:   - A lack of local policies/guidelines on effective pain management. |  |
| Cogan et al., 2014 | Canada | HIC | Qualitative cross-sectional | To evaluate patient attitudes and beliefs regarding pain medication and barriers to adequate pain management after cardiac surgery | Patients [n=379] | Patient-related:   - Fear of addiction to pain medication. - Fear of adverse side-effects. - Concerns about the ineffectiveness of prescribed analgesics. |  |
| Collada-Fernández et al., 2026 | Spain | HIC | Qualitative cross-sectional | To improve postoperative pain management in patients undergoing neurosurgery and vascular surgery in a tertiary hospital in Ciudad Real, Spain. | Patients [n=30] | Patient-related:   - Limited pre-surgery information about pain expectations and postoperative management. - Lack of adequate sleep overnight due to noisy wards.   Clinician-related:   - Lack of empathy towards patient suffering. - Fatigue due to work overload.   System-related:   - Lack of in-service training on effective pain management after surgery. - Under-reporting of postoperative pain outcomes. - Heavy workload - Disruption in continuity of pain management from the recovery room to the wards. |  |
| Dihle et al., 2006 | Norway | HIC | Qualitative, descriptive | To understand how nurses contribute to postoperative pain management in a surgical setting and to identify barriers to achieving optimal postoperative pain alleviation. | Nurses [n=9] | Clinician-related:   - Inconsistent sharing of pain-related information among clinicians. - Limited competency in conducting comprehensive pain assessments. - Lack of routine evaluation of treatment efficacy. |  |
| Eshete et al., 2019 | Ethiopia | LMIC^[2]^ | Qualitative, descriptive | To explore the views of patients, healthcare professionals, and hospital officials on barriers to and facilitators of quality pain management in Ethiopian hospitals | Patients, nurses, surgeons, anaesthetists, & gynecologists [N=24] | Patient-related:   - Positive social recognition of one's ability to handle pain. - Belief that analgesics are unnecessary during tissue healing. - Fear of addiction to pain medication. - Fear of adverse side-effects. - Limited knowledge about the surgical procedure, postoperative pain experience, and options for pain management.   Clinician-related:   - Lack of empathy towards patient suffering. - Limited proficiency in using various pain assessment tools. - Fear of legal consequences due to adverse side effects and opioid dependency.   System-related:   - A high patient-to-clinician ratio. - Limited narcotics and opioids availability due to high cost. - Postoperative pain not prioritised in healthcare. | Patient-related:   - Comprehensive information about the surgical procedure, postoperative pain experience, and options for pain management.   Clinician-related:   - Building positive rapport with patients. - Cultural competence in engaging with pain patients from diverse cultures and languages   System-related:   - In-service training on effective postoperative pain management. - Strict supervision of trainees in administering postoperative pain management. - Established guidelines/policies for quality pain management. |
| Johnson et al., 2015 | Rwanda | LMIC | Qualitative, descriptive | To determine the perspectives, perceptions and experiences of anaesthesia residents regarding postoperative pain management strategies | Anaesthetists [n=10] | Clinician-related:   - Poor communication with patients regarding pain management. - Limited hand-on experience in pain management. - Inconsistent approaches to postoperative pain assessment. - Lack of awareness of existing pain management protocols.   System-related:   - Disruption in continuity of pain management from the recovery room to the wards. - Limited resources for pain management (medication, equipment, staff) |  |
| Kidanemariam et al., 2020 | Eritrea | LMIC | Quantitative cross-sectional survey | To assess nurses’ utilisation of non-pharmacological methods in postoperative pain and the perceived barriers for their implementation at the National Hospitals. | Nurses [154] | Patient-related:   - Language barrier between patients and healthcare providers. - Uncooperative behaviour in taking pain medication.   Clinician-related:   - Limited specialised knowledge and skills in pain assessment and management. - The belief that nonpharmacological treatments are ineffective after surgery. - Belief that a nurse's primary task is to administer pain relief medication rather than contribute to a more holistic approach to pain management.   System-related:   - Lack of administrative support. - Heavy workload - Limited resources for pain management (medication, equipment, staff) - A lack of local policies/guidelines on effective pain management. |  |
| Leegaard et al 2011 | Canada | HIC | Qualitative, descriptive | To identify nurses' learning needs to prepare patients for managing pain after cardiac surgery. | Nurses [n=22] | Patient-related:   - Limited expression of pain in older adults. - Language barrier between patients and healthcare providers. - Fear of dependence and addiction to pain medication. |  |
| Makou et al 2025 | South Africa | LMIC | Qualitative, explorative, descriptive | To explore barriers that hinder the surgical wards nurses to manage acute postoperative pain at the selected public hospital in Tshwane municipality, Gauteng Province, South Africa. | Surgical Nurses [n=13] | Patient-related:   - Cultural and religious beliefs hindering pain expression. - Language barrier between patients and healthcare providers. - Tendency to report pain to the doctor but not the nurse (and other health professionals).   Clinician-related:   - Limited specialised knowledge and skills in pain assessment and management.   System-related:   - Heavy workload - Staff shortage |  |
| Maribbay et al 2023 | Saudi Arabia | HIC | Quantitative cross-sectional survey | To assess nurses' knowledge and attitude towards pain assessment and management as an essential component of holistic nursing and patient care | Nurses [n=282] | Patient-related:   - Reluctance to report pain - Reluctance to take pain medication - Fear to ask for pain medication - Tendency to report pain to the doctor but not the nurse (and other health professionals).   Clinician-related:   - Poor communication with patients regarding pain management. - Limited specialised knowledge and skills in pain assessment and management. - Limited hand-on experience in pain management. - Concerns about the addictive potential of opioids commonly used for postoperative pain management.   System-related:   - Strict regulation and access to opioids - Limited resources for pain management (medication, equipment, staff) - A lack of local policies/guidelines on effective pain management - Lack of alternative comfort measures |  |
| Martorella et al., 2021 | USA | HIC | Qualitative, descriptive | To explore the potential implementation of a nursing intervention to prevent chronic postoperative pain in the subacute phase by describing nurses' viewpoints of current barriers and facilitators. | Nurses [n=75] | System-related:  Disruption in continuity of pain management from the recovery room to the wards. |  |
| Medrzycka-Dabrowka et al., 2017 | Poland | HIC | Mixed method | To describe Polish nurses’ perceived barriers to postoperative pain in older adults. | Nurses [n=2000] | Patient-related:   - Difficulty in completing self-report pain assessment forms and scales. - Fear of addiction to pain medication. - Fear to ask for pain medication - Language barrier between patients and healthcare providers. - Difficulty assessing pain in patients with cognitive impairments. - Tendency to report pain to the doctor but not the nurse (and other health professionals).   Clinician-related:   - Physicians' distrust in nursing pain assessments. - Fear of legal consequences due to adverse side effects and opioid dependency. - Difficulty believing patient pain reports.   System-related:   - Strict regulation and access to opioids and narcotics. - Inconsistent approaches to postoperative pain assessment. - A lack of local policies/guidelines on effective pain management. - Inadequate time to conduct non-pharmacological pain treatments. - Limited opportunity to discuss the patient’s pain management with the care team. |  |
| Menlah et al 2023 | Ghana | LMIC | Quantitative cross-sectional survey | To assess the barriers and factors influencing postoperative pain management by Ghanaian nurses working in four hospitals. | Nurses [n=168] | Patient-related:   - Limited knowledge about the surgical procedure, postoperative pain experience, and options for pain management.   Clinician-related:   - Inadequate cooperation and support from physicians and pharmacists. - Limited specialised knowledge and skills in pain assessment and management. - Concerns about the addictive potential of opioids commonly used for postoperative pain management. - Difficulty believing patient pain reports. - Failure to prioritise pain management, viewing it as a normal part of surgery.   System-related:   - A lack of local policies/guidelines on effective pain management. - Limited resources for pain management (medication, equipment, staff) - Lack of in-service training on effective pain management after surgery. |  |
| Nyirigira et al., 2018 | Rwanda | LMIC | Mixed method | To identify opportunities to improve knowledge translation for postoperative pain management in Rwanda by exploring clinician and environmental factors affecting this practice. | Nurses [N=131] | Clinician-related:   - Concerns about the addictive potential of opioids commonly used for postoperative pain management. - Limited specialised knowledge and skills in pain assessment and management.   System-related:   - Limited resources for pain management (medication, equipment, staff) |  |
| Rantala et al., 2014 | Finland | HIC | Mixed method | To explore perceptions of nursing staff regarding barriers to postoperative pain management in hip fracture patients with dementia, their expectations, and facilitators offered by their employers to overcome these barriers. | Nurses [n=331] | Patient-related   - Cultural and religious beliefs hindering pain expression. - Challenges of hearing and/or visual impairments in the elderly. - Fear of dependence and addiction to pain medication. - Positive social recognition of one's ability to handle pain. - Reluctance to report pain.   Clinician-related   - Limited specialised knowledge and skills in pain assessment and management. - Concerns about the addictive potential of opioids commonly used for postoperative pain management. - Failure to document the patient’s pain treatment plan.   System-related:   - A lack of local policies/guidelines on effective pain management. - Inadequate time to conduct non-pharmacological pain treatments. - Limited resources for pain management (medication, equipment, staff) - Unclear instructions regarding the administration of requested analgesics. |  |
| Rejeh et al., 2008 | Iran | LMIC | Qualitative, descriptive | To determine Iranian nurses' perceptions of the barriers and facilitators influencing their management of postoperative pain | Nurses [n=26] | Clinician-related:   - Powerlessness to intervene independently, limited to following doctor's orders.   System-related:   - A lack of local policies/guidelines on effective pain management. | Patient-related:   - Willingness to express and report pain. - Building positive rapport with patients. - Commitment to the professional code of ethics and respect for patient rights.   System-related:   - In-service training on effective postoperative pain management. - Adopting a team approach to coordinating patient care and services. |
| Rejeh et al., 2009 | Iran | LMIC | Qualitative, descriptive | To identify nurses' experiences and perceptions about the barriers to postoperative pain management. | Nurses [n=26] | Patient-related:   - Limited pre-surgery information about pain expectations and postoperative management.   Clinician-related:   - Poor clinician-patient rapport.   System-related:   - Limited nursing authority due to physician-centred practice. - Heavy workload. |  |
| Schafheutle et al., 2001 | UK | HIC | Mixed method | To identify perceived barriers to effective pain management in nursing practice | Nurses [n=186] | Patient-related:   - Fear of injections - Aversion to taking tablets.   Clinician-related:   - Failure to assess pain based on the misconception that a patient not displaying pain-related behaviours is not in pain.   System-related:   - Limited resources for pain management (medication, equipment, staff) |  |
| Shoqirat, 2015 | Jordan | LMIC | Qualitative, descriptive | To explore patient experiences of nursing pain management in Jordan and identify contributing factors | Patients [n=31] | Patient-related:   - Fear of dependence and addiction to pain medication. - Reluctance to report pain - Limited pre-surgery information about pain expectations and postoperative management. - Not being personally known by clinical staff, leading to a lack of quality care and attention. |  |
| Shoqirat et al., 2019 | Jordan | LMIC | Quantitative cross-sectional survey | To explore the attitudes and experiences relating to opioids, in general, and morphine, in particular, among Jordanian surgical nurses. | Nurses [n=120] | Patient-related:   - Reluctance to report pain - Risk of drug addiction and/or abuse   Clinician-related:   - Limited specialised knowledge and skills in pain assessment and management. - Concerns about the addictive potential of opioids commonly used for postoperative pain management.   System-related:   - Limited access to professional pain   Specialists.   - Strict regulation and access to opioids |  |
| Temesgen et al., 2022 | Ethiopia | LMIC | Mixed method | To determine pain assessment documentation, pain management compliance with WHO guidelines, and its barrier. | Patients [n=365] | System-related:   - Strict regulation and access to opioids. - Lack of in-service training on effective pain management after surgery. - A lack of local policies/guidelines on effective pain management. - Limited resources for pain management (medication, equipment, staff) |  |
| Tomaszek et al., 2018 | Poland | HIC | Qualitative cross-sectional | To explore knowledge, compliance with good clinical practices and barriers to effective control of postoperative pain among nurses from hospitals with and without a “Hospital without Pain” certificate. | Nurses [n=500] | Clinician-related:   - Lack of empathy towards patient suffering. - Limited specialised knowledge and skills in pain assessment and management.   System-related:   - Limited resources for pain management (medication, equipment, staff). - A lack of local policies/guidelines on effective pain management. |  |
| Zachodnik et al., 2019 | Denmark | HIC | Qualitative cross-sectional | To investigate which factors influence and prevent optimal pain treatment according to healthcare providers. | Doctors,  nurses, dentists, & social and healthcare assistants [n=124] | Clinician-related:   - Limited specialised knowledge and skills in pain assessment and management.   System-related:   - Limited resources for pain management (medication, equipment, staff). - A lack of local policies/guidelines on effective pain management. - Heavy workload. |  |

1. HIC = High income country
2. Low- and Middle-income country
